# Association of Violence Against Female Sex Workers Who Use Drugs With Nonfatal Drug Overdose in Kazakhstan

**DOI:** 10.1001/jamanetworkopen.2020.20802

**Published:** 2020-10-12

**Authors:** Nabila El-Bassel, Andrea Norcini Pala, Trena I. Mukherjee, Tara McCrimmon, Gaukhar Mergenova, Assel Terlikbayeva, Sholpan Primbetova, Susan S. Witte

**Affiliations:** 1 Columbia University School of Social Work, Columbia University, New York, New York; 2 Global Health Research Center of Central Asia, Almaty, Kazakhstan; 3 Department of Epidemiology, Mailman School of Public Health, Columbia University, New York, New York

## Abstract

**Importance:**

Female sex workers (FSWs) who use drugs face increased risk of intimate partner violence (IPV) and nonpartner violence (NPV). The association between violence and drug overdose is unknown.

**Objective:**

To examine the association between IPV, NPV, and nonfatal drug overdose among FSWs who use drugs in Kazakhstan.

**Design, Setting, and Participants:**

This cross-sectional study included 400 adult FSWs in Kazakhstan who reported illicit drug use in the past year, exchanged sex for money or drugs, and reported having unprotected sex in the past 90 days. Baseline data were collected from February 2015 to May 2017 from Project Nova, an HIV prevention study among FSWs who use drugs in Kazakhstan. Data analysis was conducted from April 2019 to March 2020.

**Exposures:**

Lifetime and recent (past 90 day) experiences of physical, sexual, and psychological IPV and NPV using the Revised Conflict Tactics Scale. Subtypes of violence were identified using exploratory factor analysis.

**Main Outcomes and Measures:**

Lifetime and recent incidence of nonfatal overdose; sociodemographic characteristics as well as lifetime and recent sex work and drug use behaviors were also collected.

**Results:**

The 400 participants had a mean (SD) age of 34.1 (8.4) years. Most experienced food insecurity (358 [89.5%]) and homelessness (232 [58.0%]) in the past 90 days; one-third (130 [32.5%]) reported a history of incarceration. Most (359 [89.7%]) experienced some form of violence; 150 (37.5%) reported a lifetime nonfatal overdose, of whom 27 (18.0%) reported nonfatal overdose in the past 90 days. Lifetime severe physical violence (adjusted odds ratio [aOR], 1.27; 95% CI, 1.02-1.59; *P* = .03), engagement in sex work for more than 10 years (aOR, 2.54; 95% CI, 1.50-4.28; *P* < .001), and a history of incarceration (aOR, 4.34; 95% CI, 2.58-7.32; *P* < .001) were associated with greater odds of nonfatal overdose. Engaging in sex work for more than 10 years (aOR, 3.97; 95% CI, 1.36-11.61; *P* = .01) and a history of incarceration (aOR, 3.63; 95% CI, 1.39-9.48; *P* = .008) were associated with greater odds of recent nonfatal overdose.

**Conclusions and Relevance:**

In this study, violence against FSWs who use drugs in Kazakhstan was associated with increased odds of nonfatal overdose. Harm reduction programs for women should consider including services to address gender-based violence and the needs of women after incarceration.

## Key Points

**Question:**

Is violence against female sex workers (FSWs) who use drugs associated with an increased risk of nonfatal overdose in Kazakhstan?

**Findings:**

This cross-sectional study of 400 FSWs who use drugs found that intimate partner and nonpartner violence, especially severe physical violence, were significantly associated with experiencing nonfatal overdose. This study also found that a history of incarceration was associated with increased risk of overdose in this population.

**Meaning:**

The findings of this study suggest that harm reduction programs must consider the unique needs of women, including services to address gender-based violence and the needs of women after incarceration, to more effectively engage women in overdose prevention efforts.

## Teaser

This cross-sectional study examines the association between intimate partner violence, nonpartner violence, and nonfatal drug overdose among female sex workers who use drugs in Kazakhstan.

## Introduction

Of the 3.5 million women who inject drugs globally, approximately 1 million engage in sex work.^1^ Although overdose data are rarely disaggregated by sex or key population, studies suggest the rate of overdose mortality is increasing faster among women than men^2^ and among female sex workers (FSWs), owing to violence and drug use.^3,4^ Because women who inject drugs are outnumbered 4 to 1 by men,^5^ gender-specific needs are often overlooked, and more research examining the unique consequences of the opioid epidemic among women is needed.

The overlap between drug use and sex work is especially pronounced in Eastern Europe and Central Asia owing to the collapse of the Soviet Union.^6^ A study in Kazakhstan found that 40.3% of women who inject drugs also sold sex to support their drug habit.^7^ Moreover, the rate of nonfatal overdoses in Central Asia has been increasing, amounting to 38% of all overdoses in 2017.^8^ Overdose disproportionately affects women in Kazakhstan, where the risk of fatal overdose is 3 times higher among women who use drugs than among men who use drugs,^9^ and the proportion of women who use drugs overdosing has increased from 23.1% in 2007 to 47.2% in 2011.^10^ The true scope of the opioid overdose epidemic in Central Asia is likely underestimated owing to forced registration of people who use drugs at narcology sites and lack of data.^11^ Although women are disproportionally affected by violence and the drug use epidemic, there are considerable gaps in our understanding of the factors associated with overdose among women.^12^

Overdose among women may be driven by the physical and psychological effects of violence,^13,14,15^ which adversely affect quality of life and health outcomes. Intimate partner violence (IPV) refers to any behavior in an intimate relationship that causes physical, psychological, or sexual harm. Similarly, nonpartner violence (NPV) refers to acts of physical violence, sexual violence, emotional abuse, and/or controlling behaviors perpetrated by non–intimate partners. IPV and NPV have been consistently associated with drug use,^16^ given that victims of violence may turn to drugs to cope with psychological and physical pain, posttraumatic stress disorder, depression, and anxiety derived from experiencing violence.^17^ However, little evidence exists exploring the association between violence and overdose. The limited evidence suggests that women experiencing an overdose are also subject to opportunistic physical and sexual violence,^18^ and sexual NPV is associated with an increased risk of alcohol use disorders.^19^

FSWs are frequently subject to violence from intimate partners and non–intimate partners who include clients, pimps, police, and drug dealers.^20^ FSWs are more likely to experience multiple and overlapping types of violence, which may not be fully understood by a priori categories of violence (eg, physical, sexual, and psychological violence).^20,21^ This study advances our understanding of the association between violence from intimate as well as non–intimate partners and nonfatal overdose among women who engage in sex work and use drugs in Kazakhstan. This study aims to identify subtypes of IPV and NPV that FSWs who use drugs experience and then examine the association between violence subtypes and nonfatal overdose.

## Methods

### Participants

We used baseline, cross-sectional data from Project Nova, a cluster-randomized clinical trial of a combination HIV risk reduction and microfinance intervention in 2 cities in Kazakhstan. More information on participant recruitment and screening can be found elsewhere.^22,23^ Briefly, 763 women were recruited and screened from nongovernmental organizations, HIV and drug treatment clinics, hotels, saunas, and street-based sex work venues. Participants were also able to refer other women to the study. Women were eligible if they were older than 18 years; reported illicit drug use within the past 12 months; provided sex in exchange for money, goods, or services in the past 90 days; reported at least 1 incidence of unprotected sex within the past 90 days; were able to communicate in Russian; did not intend to move away from the study site in the following 12 months; and were not cognitively impaired. Of 410 eligible participants, 400 completed a computer-assisted self-interview baseline assessment in Russian. Verbal informed consent was obtained from all participants prior to screening, and written informed consent was obtained from all eligible study participants prior to participating in research-related activities. All screening, data collection, and intervention procedures occurred in project field offices. Study staff referred participants to sexually transmitted infection and/or drug treatment, legal services, and violence services if needed. All study protocols were approved by ethics committees at Columbia University and the Kazakhstan School of Public Health. This study was registered on ClinicalTrials.gov (NCT02406482). The reporting followed the Strengthening the Reporting of Observational Studies in Epidemiology (STROBE) reporting guideline.

### Data Collection and Measures

Baseline assessment included sociodemographic, drug use, and sex work characteristics and lifetime and recent (ie, past 90 days) experience of overdose, IPV, and NPV. Sociodemographic characteristics included age, food insecurity and homelessness in the past 90 days, marital status, educational attainment, lifetime history of incarceration, drug use history, and drug use behaviors. We also assessed years of sex work, number of clients, and other sex work–related characteristics.

To assess participant drug use, participants identified which drugs they had recreationally used ever (lifetime) and recently (in past 90 days) from a list of 24 substances that included heroin, street-based methadone, synthetic opioids, morphine, codeine, opium, tramadol, fentanyl, sedatives or tranquilizers, amphetamines, barbiturates, stimulants, and marijuana. Participants also had the opportunity to report additional illicit drug use not on the list.

To measure multiple types of IPV and NPV, we used the Revised Conflict Tactics Scale.^18^ The scale consists of 15 items that assess lifetime and recent (ie, past 90 days) experiences of physical, sexual, and psychological violence (eTable 2 in the Supplement). Participants identified the perpetrator of recent acts of violence. Violence from a current or past intimate partners was categorized as IPV. Violence from other perpetrators (eg, client, pimp, police) was categorized as NPV.

Lifetime experience of nonfatal overdose was assessed with a binary response to the question, “Have you ever overdosed on any drug?” Participants indicated which drugs they were using when they overdosed from a list of drugs. Participants with prior nonfatal overdose were asked if they experienced a nonfatal overdose in the past 90 days and what the response to their overdose was. To characterize the response to overdose, participants were asked to select all that applied from a list of actions, including calling an ambulance, receiving rescue breathing, and injecting naloxone. We also asked participants the number of fatal and nonfatal overdoses they had witnessed in the past 90 days and whether they were aware of naloxone, an opioid antagonist that rapidly reverses overdose.

### Statistical Analysis

Descriptive statistics are presented using count and proportion for categorical variables and mean and SD for continuous variables. We also assessed bivariate associations of overdose with sociodemographic characteristics and substance use and sex work behaviors. Next, we performed an exploratory factor analysis (EFA) with weighted least square estimation and tetrachoric correlation matrix on the Revised Conflict Tactics Scale items to identify subtypes of violence in this population. We tested the goodness of fit of the hierarchical model of recent experiences of IPV and NPV using exploratory structural equation modeling (ESEM) and generated factor scores using the Anderson-Rubin method for orthogonal factors.^24^ More details on EFA, ESEM, and factor scores are included in the eAppendix in the Supplement. The factor scores were then used to assess the association between subtypes of violence and overdose. Overdose and violence-related variables had no missing data. Two sex work-related variables had 7 missing values, which we accounted for in the regression analysis using full information maximum likelihood estimation.^25^

First, we examined the association between lifetime experience of violence subtypes and nonfatal overdose (ie, no prior overdose vs prior overdose). Next, we examined the association of recent experiences of IPV and NPV subtypes with overdose. We performed a multivariable penalized logistic regression analysis with Firth bias correction^26^ to account for the low prevalence of recent overdose (27 participants [1.8%]) and to improve logistic regression estimates. Penalized logistic regression models were adjusted for covariates significantly associated with experience of nonfatal overdose in bivariate analysis. These include age, years of sex work, street-based sex work, exchanging sex for services, and history of incarceration. Akaike information criterion and deviance were used to assess model fit.^27^ We defined statistical significance as a 2-tailed *P* < .05. EFA and ESEM were performed with R version 4.0.0 (R Project for Statistical Computing) package psych, and penalized logistic regression was performed with brglm2.

## Results

Among the 400 participants (Table 1), the mean (SD) age was 34.1 (8.4) years; nearly two-thirds of participants were currently or previously married (276 [69.0%]) and had children under the age of 18 years (250 [62.5%]). One-third of the women did not finish high school (127 [31.8%]) and were previously incarcerated (130 [32.5%]). Most participants reported food insecurity (358 [89.5%]) and homelessness (232 [58.0%]) in the past 90 days.

**Table 1.** Sociodemographic, Substance Use, and Sex Work Characteristics of Women Who Use Drugs and Engage in Sex Work in Kazakhstan

Characteristic	Women, No. (%)			*P* value^a^
	Total (N = 400)	Prior overdose (n = 150)	No prior overdose (n = 250)	
**Sociodemographic characteristics**				
Age, mean (SD), y	34.1 (8.4)	36.0 (7.1)	33.0 (sSs8.9)	.001
Food insecurity in past 90 d	358 (89.5)	139 (92.7)	219 (87.6)	.11
Homelessness in past 90 d	232 (58.0)	94 (62.7)	138 (55.2)	.14
Marital status				
Never married	124 (31.0)	45 (30.0)	79 (31.6)	.89
Married	106 (26.5)	39 (26.0)	67 (26.8)	
Previously married	170 (42.5)	66 (44.0)	104 (41.6)	
Did not finish high school	127 (31.8)	46 (30.7)	81 (32.4)	.72
History of incarceration	130 (32.5)	82 (54.7)	48 (19.2)	<.001
**Substance use**				
Any opioid use	276 (69.0)	138 (92.0)	138 (55.2)	<.001
Heroin	212 (53.0)	120 (80.0)	92 (36.8)	<.001
Street-based methadone	71 (17.8)	56 (37.3)	15 (6.0)	<.001
Synthetic opiates	114 (28.5)	75 (50.0)	39 (15.6)	<.001
Morphine	36 (9.0)	26 (17.3)	10 (4.0)	<.001
Codeine	46 (11.5)	33 (22.0)	13 (5.2)	<.001
Opium	71 (17.8)	52 (34.7)	19 (7.6)	<.001
Tramadol	193 (48.3)	105 (70.0)	88 (35.2)	<.001
Other opioids^b^	35 (8.8)	20 (13.3)	15 (6.0)	.04
Any sedative use	123 (30.8)	70 (46.7)	53 (21.2)	<.001
Any amphetamine use	60 (15.0)	33 (22.0)	27 (10.8)	.002
Marijuana	313 (78.3)	122 (81.3)	191 (76.4)	.25
Other drugs	176 (44.0)	90 (60.0)	86 (34.4)	<.001
Injection drug use				
Any	184 (46.0)	120 (80.0)	64 (25.6)	<.001
In past 90 d	136 (34.0)	99 (66.0)	37 (14.8)	<.001
**Sex work behaviors**				
Time engaging in sex work, mean (SD) [range], y	10.0 (7.4) [0-40]	12.5 (7.1) [0-36]	8.5 (7.1) [0-40]	<.001
Different clients in past 90 d, mean (SD) [range], No.	20.7 (39.8) [0-300]	22.0 (37.8) [0-300]	20.0 (41.0) [0-300]	.63
Street-based sex work	118 (30.0)	60 (41.4)	58 (23.4)	<.001
Ever exchanged sex for				
Money	351 (87.8)	134 (89.3)	217 (86.8)	.45
Drugs	128 (32.0)	75 (50.0)	53 (35.3)	<.001
Necessities, ie, food, clothing, place to sleep	142 (35.5)	59 (32.0)	83 (33.2)	.22
Goods, ie, jewelry, electronics	74 (18.5)	28 (40.0)	46 (18.4)	.95
Services, ie, avoiding arrest or eviction or receiving legal aid or transportation	96 (24.0)	48 (32.0)	48 (19.2)	.004

^a^ The χ^2^ or Fisher exact test was used for categorical data, *t* test for normally distributed continuous data, and Kruskal-Wallis for the variable number of different clients in past 90 days, which deviated from normal distribution (skewness, 4.27; kurtosis, 22.04).

^b^ The variable other opioids includes prescription opioids, fentanyl, desomorphine (ie, krokodil), and vint.

### Violence History

Lifetime violence and recent IPV and NPV using traditional violence categorization are reported in Table 2. Nearly all participants experienced at least some form of violence in their lifetimes (359 [89.7%]). Physical violence (350 [87.5%]) was the most common, followed by sexual violence (315 [78.8%]) and psychological violence (286 [71.5%]). In the past 90 days, more than half (206 [51.5%]) reported IPV, most commonly physical IPV (152 [38.0%]) or sexual IPV (115 [28.7%]), with fewer reporting recent psychological IPV (71 [17.7%]). Nearly half (160 [40.0%]) reported some form of NPV, from clients (113 [28.3%]), pimps (26 [6.5%]), or police (96 [24.0%]). More than one-quarter reported physical (106 [26.5%]) or sexual NPV (108 [27.0%]), and one-tenth reported psychological NPV (41 [10.3%]).

**Table 2.** Prevalence of Violence Experienced by Female Sex Workers Who Use Drugs in Kazakhstan

Type of violence	Women, No. (%)
Lifetime experiences of violence	359 (89.7)
Physical violence	350 (87.5)
Sexual violence	315 (78.8)
Psychological violence	286 (71.5)
Recent experiences of intimate partner violence	206 (51.5)
Physical violence	152 (38.0)
Sexual violence	115 (28.7)
Psychological violence	71 (17.7)
Recent experiences of nonpartner violence	160 (40.0)
Physical violence	106 (26.5)
Sexual violence	108 (27.0)
Psychological violence	41 (10.2)

### Nonfatal Overdose

More than one-third of participants (150 [37.5%]) experienced a nonfatal overdose in their lifetime, 27 (18.0%) of whom experienced a nonfatal overdose in the past 90 days. Most participants experienced a nonfatal overdose from heroin (113 [75.3%]) (Table 3). Few (41 [10.3%]) were aware of Naloxone.

**Table 3.** Prevalence of Nonfatal Overdose Experienced and Witnessed and Overdose-Related Characteristics

Factor	No./total No. (%)
Ever experienced nonfatal overdose	150/400 (37.5)
Substance overdosed while using	
Heroin	113/150 (75.3)
Other	37/150 (24.7)
Heard of naloxone	41/400 (10.3)
Overdose in past 90 d	27/150 (18.0)
Response to past 90-day overdose	
Called ambulance	6/27 (22.2)
Received rescue breathing	9/27 (33.3)
Injected saline solution	11/27 (40.7)
Injected naloxone	2/27 (7.4)
Other^a^	9/27 (33.3)
Non-fatal overdoses witnessed in past 90 d among all women, mean (SD) [range], No.	2.8 (6.0) [0-60]
Fatal overdoses witnessed in past 90 d among 171 women, mean (SD) [range], No.	2.9 (4.7) [0-30]

^a^ Other options include received emergency medical care, injected cardiamine, had their chest rubbed.

### Characteristics Associated With Lifetime Nonfatal Overdose

More than two-thirds of women (276 [69.0%]) reported recently using any opioids, with heroin (212 [53.0%]) and tramadol (193 [48.3%]) use being the most common (Table 1). Older women, those with a history of incarceration, and those who reported injection drug use were more likely to experience overdose (prior overdose vs no prior overdose: mean [SD] age, 36.0 [7.1] years vs 33.0 [8.9] years; *P* < .001; history of incarceration, 82 [54.7%] vs 48 [19.2%]; *P* < .001; injection drug use, 120 [80.0%] vs 64 [25.6%]; *P* < .001). Women who had engaged in sex work longer, exchanged sex for drugs, or were engaged in street-based sex work were also more likely to experience overdose (prior overdose vs no prior overdose: mean [SD] years of sex work, 12.5 [7.1] years vs 8.5 [7.1] years; *P* < .001; exchanged sex for drugs, 75 [50.0%] vs 53 [35.3%]; *P* < .001; engaged in street-based sex work, 60 [41.4%] vs 58 [23.4]; *P* < .001).

### Associations Between Lifetime Violence Subtypes and Nonfatal Overdose

The EFA resulted in 3 factors, measuring 3 subtypes of violence that were not intercorrelated, as follows: multiple types of violence, severe physical violence, and sexual coercion (eTable 1 and eTable 2 in the Supplement). The subtypes of violence consist of unique combinations of co-occurring psychological, physical, and sexual violence. Because the 3 subtypes were not intercorrelated (ie, orthogonal rotation), each subtype identified independent experiences of violence. For example, a woman who scored high on the multiple types of violence subtype has experienced severe and moderate physical, sexual, and psychological violence. If she also scores high on severe physical violence, she has been further exposed to instances of severe physical violence.

Multiple types of violence and severe physical violence subtypes were associated with 76% and 56% increased odds of nonfatal overdose in the unadjusted model (multiple types of violence: adjusted odds ratio [aOR], 1.76; 95% CI, 1.35-2.28; *P* < .001; severe physical violence: aOR, 1.56; 95% CI, 1.06-1.58; *P* < .001) (Table 4). After adjusting for covariates, lifetime experience of multiple types of violence was not associated with a 34% increase of nonfatal overdose (aOR, 1.34; 95% CI, 0.99-1.81; *P* = .05), whereas women who experienced severe physical violence had a 27% higher odds of prior overdose (aOR, 1.27; 95% CI, 1.02-1.59; *P* = .03). Additionally, women with a history of incarceration and those who had engaged in sex work for more than 10 years had 4.34-fold (95% CI, 2.58-7.32; *P* < .001) and 2.54-fold (95% CI, 1.50-4.28; *P* < .001) increased odds of nonfatal overdose, respectively.

**Table 4.** Association of Lifetime Experience of Subtypes of Violence With Nonfatal Overdose, Penalized Logistic Regression Analysis

Factor	Prior overdose	
	Unadjusted OR (95% CI)	Adjusted OR (95% CI)^a^
Multiple types of violence, ever	1.76 (1.35-2.28)^b^	1.34 (0.99-1.81)^c^
Severe physical violence, ever	1.56 (1.06-1.58)^d^	1.27 (1.02-1.59)^e^
Sexual coercion, ever	1.09 (0.92-1.29)	0.97 (0.80-1.17)
Age	NA	0.99 (0.95-1.02)
≥10 y of sex work	NA	2.54 (1.50-4.28)^b^
Street-based sex work	NA	1.44 (0.86-2.39)
Exchange sex for services^f^	NA	1.14 (0.83-1.56)
History of incarceration	NA	4.34 (2.58-7.32)^b^
AIC	512.8	445.9
Deviance	504.8	425.9

Abbreviations: AIC, Akaike information criterion; NA, not applicable; OR, odds ratio.

^a^ Adjusted models adjust for age, years of sex work, street-based sex work, exchanging sex for services and incarceration history.

^b^ *P* < .001.

^c^ *P* < .10.

^d^ *P* < .01.

^e^ *P* < .05.

^f^ Services include avoiding arrest or eviction or receiving legal aid or transportation.

### Associations Between Recent IPV and NPV Subtypes and Nonfatal Overdose

In the unadjusted models, recent experiences of multiple types of IPV and NPV and severe physical IPV and NPV were associated with increased odds of recent overdose (Table 5; eTable 3 in the Supplement). After adjusting for covariates, multiple types of IPV (aOR, 1.47; 95% CI, 0.97-2.23; *P* = .07) and severe physical IPV (aOR, 1.34; 95% CI, 0.95-1.88; *P* = .09) were not associated with an increased odds of nonfatal overdose. Severe physical NPV (aOR, 1.29; 95% CI, 1.00-1.67; *P* = .05) was not associated with an increased odds of recent overdose. The odds of recent overdose remained significantly higher among FSWs who engaged in sex work for more than 10 years (IPV: aOR, 4.08; 95% CI, 1.41-11.81; *P* = .009; NPV: aOR, 3.97; 95% CI, 1.36-11.61; *P* = .01) and those with history of incarceration (IPV: aOR, 4.72; 95% CI, 1.79-12.42; *P* = .002; NPV: aOR, 3.63; 95% CI, 1.39-9.48; *P* = .009).

**Table 5.** Association of Recent IPV and NPV Subtypes With Recent Nonfatal Overdose, Penalized Logistic Regression Analysis

Factor	Recent IPV		Recent NPV	
	Unadjusted OR (95% CI)	Adjusted OR (95% CI)^a^	Unadjusted OR (95% CI)	Adjusted OR (95% CI)^a^
Recent multiple types of violence	1.46 (1.00-2.13)^b^	1.47 (0.97-2.23)^c^	1.73 (1.19-2.51)^d^	1.35 (0.90-2.03)
Recent severe physical violence	1.37 (1.00-1.88)^b^	1.34 (0.95-1.88)^c^	1.35 (1.08-1.69)^d^	1.29 (1.00-1.67)^b^
Sexual coercion	0.97 (0.73 -1.30)	0.76 (0.56-1.06)	1.24 (0.91-1.69)	1.30 (0.92-1.83)
Age	NA	0.96 (0.90-1.03)	NA	0.96 (0.90-1.03)
≥10 years of sex work	NA	4.08 (1.41-11.81)^e^	NA	3.97 (1.36-11.61)^d^
Street-based sex work	NA	1.60 (0.64-3.98)	NA	2.23 (0.84-5.91)
Exchange sex for services^f^	NA	2.08 (0.79-5.46)	NA	1.29 (0.51-3.26)
History of incarceration	NA	4.72 (1.79-12.42)^d^	NA	3.63 (1.39-9.48)^d^
AIC	180.9	155.9	170.81	155.13
Deviance	172.9	135.9	162.81	135.13

Abbreviations: AIC, Akaike information criterion; NA, not applicable; OR, odds ratio.

^a^ Models were adjusted for age, years of sex work, street-based sex work, exchanging sex for services, and history of incarceration.

^b^ *P* < .05.

^c^ *P* < .10.

^d^ *P* < .01.

^e^ *P* < .001.

^f^ Services include avoiding arrest or eviction or receiving legal aid or transportation.

## Discussion

To our knowledge, this is the first study to examine the association of IPV and NPV with overdose among FSWs who use drugs. Additionally, it adds to the limited evidence of violence and overdose among women who use drugs in Central Asia. Our finding that half of our sample experienced recent IPV is consistent with prior studies that have shown that the prevalence of violence against women who use drugs is 3 times higher than against women in the general population in the United States and globally.^20,28,29^ More than two-thirds of the women who overdosed did so as a result of heroin use, similar to previous reports among women who use or inject drugs in Kazakhstan and Central Asia.^9,10,30^ Our findings are also consistent with global trends of increasing nonmedical use of pharmaceutical opioids, given that nearly half of all women in this study reported tramadol use.^31^

Our results suggest that severe physical violence from intimate and non–intimate partners is associated with an increased burden of nonfatal overdose, and urgent violence prevention interventions are needed for this high-risk population. To date, there are no specific interventions focused on reducing violence against FSWs in Central Asia. Structural approaches for HIV prevention among FSWs have been shown to reduce NPV from clients and police in India.^32,33,34^ A study of FSW and their intimate partners at the US-Mexico border also suggests that couples-based interventions that acknowledge gender power differentials, interpersonal dynamics, communication skills, coping skills (apart from drug use), or economic interventions that target the structural drivers of IPV may reduce the prevalence and severity of violence.^35^ Couples-based interventions have been found to be particularly beneficial for perpetrators of violence struggling with alcohol and substance use disorders, and have better retention compared with violence interventions for individuals.^36^

In addition to violence, our study shows that FSWs who have engaged in sex work longer and have a history of incarceration are at a higher risk of nonfatal overdose, highlighting structural factors that may contribute to overdose. Prior studies from the United States suggest that the risk of overdose is highest in the 2 weeks postrelease and that this risk is especially pronounced for women who use drugs.^37,38^ This fact, together with the high prevalence of homelessness and food insecurity among this population, highlight the urgent need for psychosocial services for FSWs that address violence and substance use. Because men who use drugs outnumber women who use drugs,^5^ treatment programs often fail to consider the gendered aspects of drug use. This lack of focus on the needs of women leads to inadequate care, and women are often excluded from essential services in Kazakhstan and other countries because of fear of losing their children, being registered with the government, or being forced into compulsory drug treatment or prison when seeking treatment.^39^ To effectively engage women in overdose prevention, harm reduction programs must take a human rights approach and provide integrated service delivery that considers gender-based violence, pregnancy, mental health, childcare, and postrelease services.^39^ Decriminalization of sex work could also lead to reductions in violence and incarceration,^40^ and as a result, overdose.

It is estimated that 20% to 50% of all women who inject drugs are involved in sex work in the former Soviet Union countries,^21^ and IPV, NPV, and opioid use disproportionately affect FSWs. Substance use treatment programs often fail to incorporate gendered perspectives on issues such as violence, trauma, incarceration, sex work, and housing,^41^ and women accessing treatment face an increased risk of physical and sexual violence and stigma.^42,43^ The lack of trauma-informed care in traditional treatment programs designed for men makes women less likely to engage in care.^44^

### Strengths and Limitations

This study used a novel data-driven approach to examine how multiple forms of violence are associated with increases in the risk of nonfatal overdose. Prior studies have shown that experiencing 1 form of violence places individuals at a higher risk of experiencing other forms of violence.^45^ However, traditional violence research tends to report individual forms of violence that are categorized as physical, psychological, or sexual violence, which fail to capture the full extent of violence as a multifaceted construct.^46^ Moreover, definitions of what constitutes violence varies by context and culture, and the dimensions of women’s experiences (ie, violence in the context of sex work or drug use) are not considered.^46^ By using a data-driven approach, we identified subtypes of co-occurring violence rather than relying on a priori categories of physical, sexual, and psychological violence. This approach allowed us to consider how multiple forms of violence cluster to represent the experiences of each participant and allowed us to distinguish between the overall consequences of experiencing multiple types of violence from the unique associations of experiencing severe physical violence and sexual coercion with nonfatal overdose.

This study is not without limitations. Because data were self-reported, social desirability, recall, and information bias are possible. Failure to include women who died as a result of experiencing severe physical violence or fatal overdose may have resulted in survivor bias. Because of the cross-sectional design, reverse causality is possible. Future studies should examine the association between violence and overdose using longitudinal data. Finally, nonrandom sampling and multiple recruitment techniques may have resulted in selection bias, which limits the generalizability of findings to other FSW populations in Central Asia or globally. Given the dearth of information available regarding the association between violence and overdose, particularly among FSWs who use drugs, we encourage further research on the mechanisms that associate violence with nonfatal overdose in other global settings.

## Conclusions

The findings if this study have important implications for harm reduction programs for women who use drugs. Currently, Kazakhstan does not have any existing overdose prevention programs for people who use drugs. In light of our findings, future harm reduction programs should consider integrating trauma-informed medical and social services for women who use drugs.

## Notes

Supplement. **eAppendix.** Supplemental Methods **eTable 1.** Subtypes of Violence, Exploratory Factor Analysis **eTable 2.** Factorial Model of the Subtypes of Violence: Exploratory Factor Analysis **eTable 3.** Recent IPV and GBV 3-Factor Models: Exploratory Structural Equation Model (ESEM) **eReferences.**
