# Supplementary material for: Association of Violence Against Female Sex Workers Who Use Drugs With Nonfatal Drug Overdose in Kazakhstan
Source: JAMA Netw Open. 2020 Oct 12;3(10):e2020802. doi: 10.1001/jamanetworkopen.2020.20802 (PMC7550967; doi:10.1001/jamanetworkopen.2020.20802)
Supplement: Supplement. — eAppendix. Supplemental Methods eTable 1. Subtypes of Violence, Exploratory Factor Analysis eTable 2. Factorial Model of the Subtypes of Violence: Exploratory Factor Analysis eTable 3. Recent IPV and GBV 3-Factor Models: Exploratory Structural Equation Model (ESEM) eReferences. [file jamanetwopen-e2020802-s001.pdf]

Supplemental Online Content

El-Bassel N, Norcini Pala A, Mukherjee TI, et al. Association of violence against female sex workers who use drugs with nonfatal drug overdose in Kazakhstan. *JAMA Netw Open*. 2020;3(10):e2020802. doi:10.1001/jamanetworkopen.2020.20802

eAppendix. Supplemental Methods
eTable 1. Subtypes of Violence, Exploratory Factor Analysis
eTable 2. Factorial Model of the Subtypes of Violence: Exploratory Factor Analysis
eTable 3. Recent IPV and GBV 3-Factor Models: Exploratory Structural Equation Model (ESEM)
eReferences.

This supplemental material has been provided by the authors to give readers additional information about their work.

© 2020 El-Bassel N et al. *JAMA Network Open.*

eAppendix. Supplemental Methods

Exploratory Factor Analysis
To identify subtypes of lifetime experiences of violence, we performed Exploratory Factor Analysis (EFA) using weighted least square mean and variance adjusted (WLS) and tetrachoric correlation matrix. We compared goodness-of-fit (GOF) indices of 1-, 2-, and 3-factor, selecting the factorial model associated with the best GOF indices and factor interpretability. GOF indices included: χ^2 statistic, Comparative Fit Index (CFI), Tucker-Lewis Index (TLI), and Root Mean Square Error of Approximation (RMSEA). Indices' cut-offs were non-significant χ^2 ($p>0.05$); CFI/TLI ≥ 0.95; and RMSEA ≤ 0.05.[1]

The 3-factor model was associated with optimal GOF indices, particularly statistically non-significant χ^2 (eTable 1). Factor loadings for each item was examined (eTable 2), and items were allowed to cross-load onto factors if they loaded >0.30. Specifically, we identified a hierarchical model, which consists of a general factor and two group factors. The general factor accounts for most of all items' variance, namely all items loaded onto the general factor. Group factors explain the items' residual variance unexplained by the general factor. Subsequently, we tested whether the same lifetime violence subtypes (i.e., 3-factor hierarchical model) applied to recent experiences of violence, IPV and GBV using Exploratory Structural Equation Modeling (ESEM; see eTable 3). ESEM is a flexible method combining confirmatory and exploratory factor analysis.[2] While confirmatory factor analysis sets cross-loadings to 0, ESEM allows cross-loadings to be approximately 0. Cross-loadings set to 0 in confirmatory factor analysis negatively affects estimation of the factors and inflates factor inter-correlation. These issues are addressed through ESEM.

Hierarchical factorial model
Through EFA, we identified factors that quantify the probability of co-occurrence of different violence experiences (see Table 2). The Multiple types of violence factor accounts for the largest proportion of co-occurrence of all forms of violence. The other two factors account for the co-occurrence probability of specific subtypes of violence (i.e. Severe physical violence and Sexual coercion), which is not explained by the first factor. For example, because all violence experiences load onto the Multiple types of violence factor, a higher score on this factor indicates a higher probability of experiencing psychological, physical, and sexual forms of violence. A higher score on the Severe Physical Violence factor suggests a higher probability of co-occurring physical violence experience. Other studies used a similar approach to investigate diverse types of violence (e.g. IPV, microaggression).[3]

Factor Scores
We generated factor scores for two main reasons. The first is that the hierarchical model cannot be captured using raw scores (i.e., sum of each violence event). The identified factors quantify the probabilities of co-occurrence of all types of violence or two specific subtypes (physical and sexual). Factor scores are representations of the probability of experiencing a given violence experience. Secondly, unlike factor scores, raw scores do not account for measurement error, namely the proportion of variability due to random or systematic fluctuations/bias. The generation of factor scores and subsequent analysis through other statistical techniques is a

© 2020 El-Bassel N et al. *JAMA Network Open.*

common procedure.[4] Factor scores quantify the probabilities of violence experiences, which are predictors of OD in the multinomial logistic regression.

© 2020 El-Bassel N et al. *JAMA Network Open.*

eTable 1. Subtypes of Violence, Exploratory Factor Analysis

Factorial models	χ^2	df	p-values	RMSEA	CFI	TLI
1-factor	233.19	90	<0.001	0.06	0.98	0.97
2-factor	100.29	76	0.030	0.03	1.00	0.99
3-factor	71.52	63	0.220	0.02	1.00	1.00

RMSEA: Root mean square of approximation; CFI: Comparative Fit Index; TLI: Tucker-Lewis Index

© 2020 El-Bassel N et al. *JAMA Network Open.*

eTable 2. Factorial Model of the Subtypes of Violence: Exploratory Factor Analysis

	**Multiple types violence**	**Severe physical violence**	**Sexual coercion**
Has anyone ever kicked you, slammed you against a wall, beaten you up, punched or kicked you, hit you with something that could hurt or burned or scalded you on purpose?	**0.68**	**0.59**	0.04
Did anyone ever break your bones, give you cuts, bruises or other injuries that required medical care because of a fight?	**0.67**	**0.54**	0.02
Has anyone ever choked you or used or threatened to use a knife or gun on you?	**0.68**	**0.51**	0.09
Has anyone ever prevented you from seeing family or friends, held you captive, stalked you, or verbally threatened to hurt you or your family?	**0.67**	**0.39**	-0.14
Has anyone ever twisted your arm, or thrown something at you that could hurt, or pushed, grabbed or slapped you?	**0.74**	**0.32**	0.13
Has anyone ever caused a permanent physical disfigurement of your body?	**0.64**	**0.30**	-0.07
Has anyone ever insisted you have sex even though you didn't want to?	**0.83**	0.15	**0.41**
Has anyone ever forced you to have sex without a condom?	**0.72**	0.05	**0.40**
Has anyone ever used force or threatened to use force to have sex?	**0.89**	0.05	**0.37**
Has anyone ever used force or threatened to use force to make you have sex with other men in exchange for money or drugs?	**0.74**	-0.10	**0.32**
Has anyone ever kept you isolated in a room (e.g. kept you from leaving), against your will?	**0.87**	0.12	-0.20
Has anyone ever deprived you of food, water, or sleep?	**0.73**	0.18	-0.18
Has anyone ever penetrated your vagina or anus with an object?	**0.71**	-0.17	0.09
Has anyone ever forced you to remove or stripped off your clothing?	**0.83**	0.04	0.10
Has anyone ever tied up or blindfolded you?	**0.66**	-0.07	-0.04

Geomin orthogonal rotation; In bold, the factor loadings greater than 0.3

© 2020 El-Bassel N et al. *JAMA Network Open.*

eTable 3. Recent IPV and GBV 3-Factor Models: Exploratory Structural Equation Model (ESEM)

		χ^2	df	p-value	CFI	TLI	RMSEA
	3-factor model - Recent IPV	69.291	63	0.270	1.00	0.99	0.022
	3-factor model - Recent GBV	53.919	63	0.790	1.00	1.00	<0.001

RMSEA: Root mean square of approximation; CFI: Comparative Fit Index; TLI: Tucker-Lewis Index; IPV: Intimate partner violence; GBV: Gender-based violence

© 2020 El-Bassel N et al. *JAMA Network Open.*

eReferences.

1. Schermelleh-Engel K, Moosbrugger H, Müller H. Evaluating the Fit of Structural Equation Models:Tests of Significance andDescriptive Goodness-of-Fit Measures. *Methods of Psychological Research Online.* 2003;8:23-74.
2. Asparouhov T, Muthén B. Exploratory structural equation modeling. *Structural equation modeling: a multidisciplinary journal.* 2009;16(3):397-438.
3. Gracia E, Rodriguez CM, Martín-Fernández M, Lila M. Acceptability of family violence: Underlying ties between intimate partner violence and child abuse. *Journal of interpersonal violence.* 2017:0886260517707310.
4. DiStefano C, Zhu M, Mindrila D. Understanding and using factor scores: Considerations for the applied researcher. *Practical Assessment, Research, and Evaluation.* 2009;14(1):20.
